# A Systems Genetics Approach Identified *GPD1L* and its Molecular Mechanism for Obesity in Human Adipose Tissue

**DOI:** 10.1038/s41598-017-01517-6

**Published:** 2017-05-11

**Authors:** Hao He, Dianjianyi Sun, Yong Zeng, Ruifeng Wang, Wei Zhu, Shaolong Cao, George A. Bray, Wei Chen, Hui Shen, Frank M. Sacks, Lu Qi, Hong-wen Deng

**Affiliations:** 1 Center for Bioinformatics and Genomics, Department of Biostatistics and Bioinformatics, Tulane University School of Public Health and Tropical Medicine, New Orleans, LA 70112 USA; 2 Tulane University Obesity Research Center, Tulane University School of Public Health and Tropical Medicine, New Orleans, LA 70112 USA; 3 Department of Biomedical Engineering, Tulane University, New Orleans, LA 70118 USA; 4 Pennington Biomedical Research Center, Louisiana State University, Baton Rouge, LA 70808 USA; 5 Department of Epidemiology, Tulane University School of Public Health and Tropical Medicine, New Orleans, LA 70112 USA; 6 Department of Nutrition, Harvard T.H. Chan School of Public Health, Boston, MA 02115 USA; 7 Department of Nutrition, Harvard T.H. Chan School of Public Health, Boston, MA Channing Division of Network Medicine, Department of Medicine, Brigham and Women’s Hospital and Harvard Medical School, Boston, MA 02115 USA

## Abstract

To explore novel molecular mechanisms underlying obesity, we applied a systems genetics framework to integrate risk genetic loci from the largest body mass index (BMI) genome-wide association studies (GWAS) meta-analysis with mRNA and microRNA profiling in adipose tissue from 200 subjects. One module was identified to be most significantly associated with obesity and other metabolic traits. We identified eight hub genes which likely play important roles in obesity metabolism and identified microRNAs that significantly negatively correlated with hub genes. This module was preserved in other three test gene expression datasets, and all hub genes were consistently downregulated in obese subjects through the meta-analysis. Gene *GPD1L* had the highest connectivity and was identified a key causal regulator in the module. Gene *GPD1L* was significantly negatively correlated with the expression of miR-210, which was experimentally validated that miR-210 regulated *GPD1L* protein level through direct interaction with its mRNA three prime untranslated region (3′-UTR). *GPD1L* was found to be upregulated during weight loss and weight maintenance induced by low calorie diet (LCD), while downregulated during weight gain induced by high-fat diet (HFD). The results indicated that increased *GPD1L* in adipose tissue may have a significant therapeutic potential in reducing obesity and insulin resistance.

## Introduction

Obesity is a complex condition characterized by excessive storage of energy mainly as lipids in white adipocytes, as a result of a positive energy balance. Epidemic of obesity has become an exponentially growing public health problem associated with several severe diseases such as type 2 diabetes (T2D), cardiovascular diseases (CVD) and various types of cancers^1^. According to a World Health Organization (WHO) report, obesity has reached epidemic proportions globally, with at least 2.8 million people dying each year from obesity.

Body mass index (BMI) is the most widely used measure for assessing obesity, and has a strong genetic component (40% to 70% heritability)^2, 3^. The largest genome-wide association study (GWAS) meta-analysis for BMI, including a total of 339,224 individuals, identified 97 obesity-associated loci (*P* < 5 × 10^−8^), which accounted for only 2.7% of BMI variation^4^. A large proportion of the genetic variance remained to be discovered. It is extremely difficult to follow up on every gene in the functional experiment among a large number of loci identified by GWAS. Therefore, prioritization of the promising genes was of great interest by placing them in a broad context such as implicated tissues/cell-types, pathways and inter-connected networks, as gene functions are commonly dependent on their tissue context and the disordered interplay of tissue/cell-type with specific processes^5^. Transcriptomic analysis on thousands of genes simultaneously can provide further insight into the molecular mechanisms of complex traits, as gene expression provides an important link between genetic variations and their corresponding phenotypic alterations. It is well recognized that network analysis is capable of revealing comprehensive transcriptional regulation for complex diseases, since genes interact with each other in complex regulatory networks. For example, gene coexpression networks comprised of modules of genes demonstrating high levels of co-regulation^6^.

**Figure 1:**
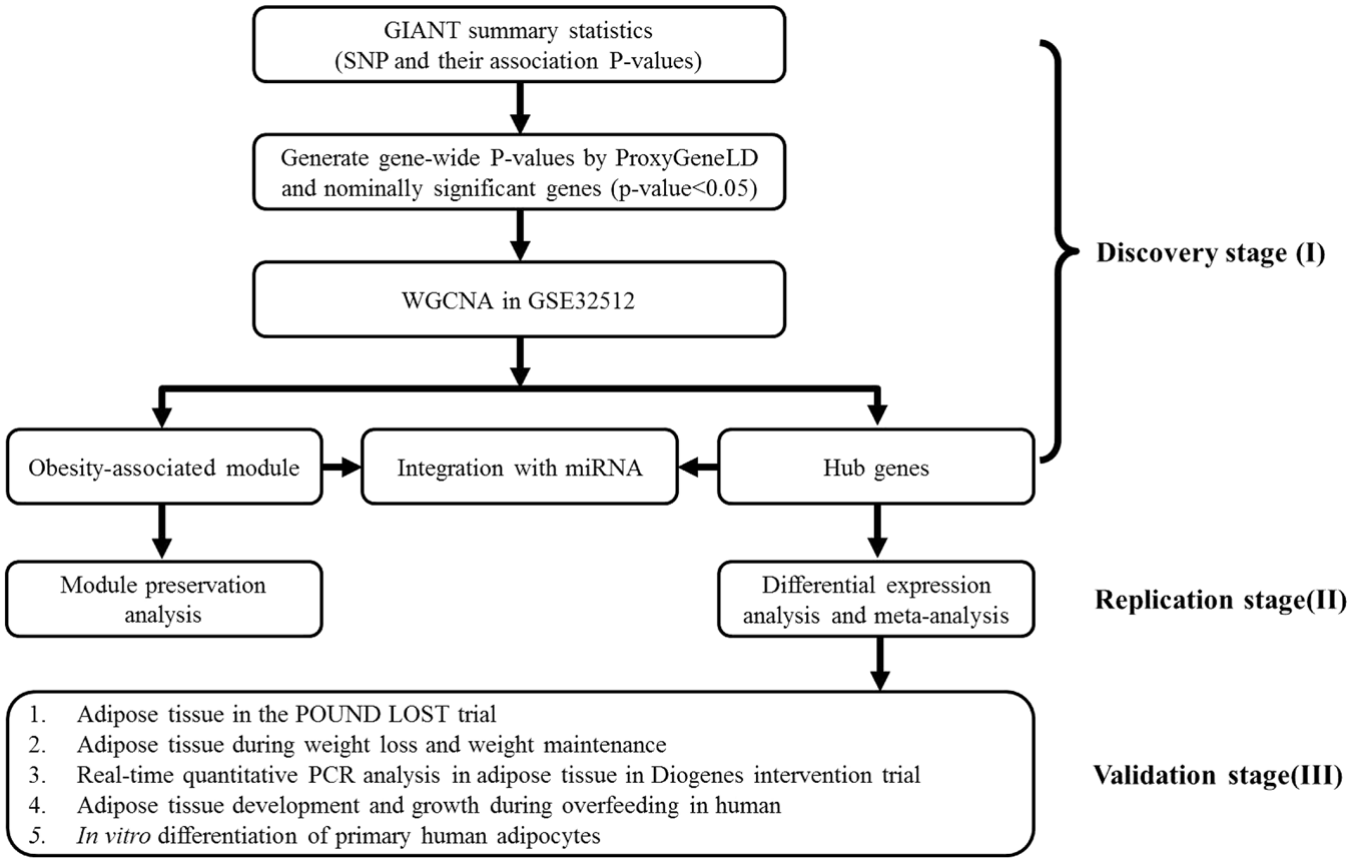
Systems genetics framework for underlying molecular mechanisms of obesity. A three-stage approach was applied.

We implemented a systems genetics framework to integrate genetic loci identified from the largest BMI GWAS meta-analysis with gene expression profiles in adipose tissue from 200 subjects to explore underlying molecular mechanisms for obesity (Fig. 1). Genetic loci associated with diseases from GWAS can perturb specific parts of gene networks, whose overall dysregulation may shift homeostatic processes and lead to disease^4, 7, 8^. Using weighted gene coexpression network analysis (WGCNA), we identified modules composed of genes that were highly interconnected with one another and displayed significant associations with obesity and related metabolic traits. Through characterization of module content and topology, we identified hub genes which likely play an important role in obesity metabolism. Next, we applied the causal network inference and key driver analysis (KDA) to identify the key causal regulators in the obesity-associated module. As adipose microRNAs (miRNAs) have been linked to adipogeneisis *in vitro* and in animal obesity models, they play an important role in the development of obesity^9^. The cooperative and combinatorial targeting ability of miRNA allow precise and robust gene regulation at both the single-gene and gene-network levels^10^. In order to assess the role of miRNA in regulating gene expression in pathological processes, we studied the regulation of human adipose miRNAs expression and their relationships with obesity-associated modules and hub genes in the same 200 subjects. Furthermore, we utilized four publicly available experiments in adipose tissues from obese (BMI > 30) and non-obese subjects to evaluate preservation of module topology. In addition, we further utilized five studies including four longitudinal dietary intervention studies (one in-house clinical trial, The POUND LOST Trial) in human abdominal subcutaneous adipose tissue to provide supportive biological information for future functional validation of hub genes’ involvement in adipose tissue metabolism.

Our goal is to better understand the biological mechanisms underlying obesity risk and to identify most important candidate causal genes for obesity by integration of genetic loci, transcriptome in adipose tissue, phenotypic data and experiments relevant to adipose tissue metabolism. A most promising candidate causal gene *GPD1L* was highlighted. Our findings provided strong support for the important role of *GPD1L* and also shed light on its molecular functional mechanisms in the etiology of obesity and insulin resistance.

## Materials and Methods

### Discovery Stage (I)

#### Gene-based method on summary statistics of BMI meta-analysis GWAS

We downloaded publicly available summary statistics from a meta-analysis of 125 studies of BMI performed by the Genetic Investigation of ANthropometric Traits (GIANT) Consortium, a total sample of 339,224 individuals (http://www.broadinstitute.org/collaboration/giant/index.php/GIANT_consortium_data_files)^4^. GWAS meta-analysis results were corrected for genomic control using all the SNPs^11^.

All the SNPs in the BMI meta-analysis GWAS were converted to gene lists using ProxyGeneLD, which calculated gene-wide p-value for each gene by reading the SNP list and BMI association statistics^12^. Based on CEU HapMap samples, ProxyGeneLD identified clusters of GWAS SNPs in high LD (*r* ^2^ ≤ 0.80). Genes were defined as the transcript plus a 1-kbp extension upstream to include promoter regions. Gene-wide p-values were then calculated as the minimum of any SNP in the gene, either as a singleton or member of a proxy cluster per gene. Genes with gene-wide *P* ≤ 0.05 for BMI were referred to as the nominally significant GWAS genes for subsequent analyses.

#### Gene expression data processing

To generate gene coexpression networks, we used a microarray data set from 200 random human subjects who were part of population-based cross-sectional Metabolic Syndrome in Men (METSIM) study^13^. Participants of the METSIM study had been extensively characterized for physical and metabolic traits, including BMI, fat mass (FM), waist-to-hip ratio (WHR), total cholesterol (TC), high-density lipoprotein (HDL), low-density lipoprotein (LDL), total triglyceride (TG) levels, adiponectin, C-reactive protein (CRP), Matsuda index of insulin sensitivity^14^ and the homeostasis model assessment-estimated insulin resistance (HOMA-IR)^15^. Total RNA from the METSIM subjects was isolated from abdominal subcutaneous adipose tissue, which was obtained with needle biopsies. High-quality RNA samples from each METSIM subject were hybridized to Illumina Human HT-12 v3 Expression Bead-Chips (San Diego, CA, USA). Data were downloaded from the National Center for Biotechnology Information Gene Expression Omnibus (GEO) (accession number: GSE32512) and subjected to a stringent quality control analysis, using non-parametric background correction followed by quantile normalization with both control and expression probes utilizing the *neqc* function in the Linear Models for Microarray Analysis (LIMMA) package in R/Bioconductor^16, 17^. Probes with detection p-value ≥0.01 in ≥90% of 200 subjects were removed, which were not significantly expressed above the background level. The remaining missing data were imputed using the k-nearest neighbor procedure, as implemented in the impute R package^18^. As part of quality-control protocol, a clustering and principal-components analysis (PCA) was performed to identify potential outliers in the dataset by using functions *hclust* and *prcomp* in R. After clustering samples based on global expression values and PC1, one sample was identified as an outlier and therefore removed from subsequent analyses.

#### Construction of coexpression networks and identification of obesity-associated gene coexpression modules

Gene coexpression networks were constructed using WGCNA^6, 9^. WGCNA is a well-established tool for studying biological networks based on pair wise correlation between variables in high-dimensional microarray data sets. First, we identified probes assaying the expression of nominally significant GWAS genes. For these genes, we then calculated Pearson correlation coefficients for all gene-gene comparisons across microarray samples. The matrix of correlations was then converted to an adjacency matrix of connection strengths. The adjacencies were defined as ${a}_{ij}={|cor({x}_{i},{x}_{j})|}^{\beta }$, where *x*_*i*_ and *x*_*j*_ are the ith and jth gene expression traits. The parameter β of the power function was defined in such a way that the resulting coexpression network (adjacency matrix) satisfies approximate scale-free topology, as previously outlined by Zhang and Horvath^19^. We chose a power of 6, which resulted in an approximate scale-free topology network with the scale-free fitting index R^2^ greater than 0.8.

Modules were defined as sets of genes with high topological overlap. The topological overlap measure (TOM) $TO{M}_{ij}=\frac{{\sum }_{u\ne i}{a}_{iu}{a}_{uj}+{a}_{ij}}{\min ({k}_{i},{k}_{j})+1-{a}_{ij}}$, where ${k}_{i}={\sum }_{u\ne i}{a}_{ui}$ denotes the network connectivity, calculated as the sum of the connection strengths with all other module genes. A TOM-based dissimilarity measure (1 − TOM) was used for hierarchical clustering. Gene modules corresponded to the branches of the resulting dendogram and were precisely defined using the “dynamic hybrid” branch-cutting algorithm^20^. Module eigengenes (MEs) were defined as the first principal component calculated using PCA, which can summarize modules’ behavior. These MEs were tested for association with obesity and other phenotypes adjusted for age using a linear regression model. Gene significance (GS) for the each module gene was defined as the absolute value of its Pearson correlation with obesity. The module membership (MM) (also referred as intramodular connectivity) of gene *i* in module *q*, kME value, was defined as the absolute value of Pearson correlation between its gene expression and the module eigengene. Specifically, $kM{E}_{q}(i)=|cor({x}_{i},M{E}_{q})|$, where *ME*_*q*_ is the module eigengene of the module *q*. Note that it specifies how close gene *i* is to module *q* and does not require that gene *i* to be a member of the module *q*. Both GS and MM can be combined in a systems biological screening method for finding hub genes, which are highly interconnected nodes within gene coexpression modules and have been shown to play key roles in controlling module behavior and disease mechanisms^6, 21, 22^. The module hub gene was defined as the gene in the module with the highest connectivity, or based on a high intra-modular connectivity (MM > 0.8) and high GS (>0.2).

To assess the importance of hub genes, we used the Mouse Genome Informatics (MGI) web tool (http://www.informatics.jax.org/)^23^ to analyze the reported endogenous gene expression during mouse development. To determine whether the obesity-associated modules were biologically meaningful, GO enrichment analyses were conducted using the WebGestalt web site (http://genereg.ornl.gov/webgestalt)^24^. We examined enrichment of Biological Process GO terms using hypergeometric test and the p-value was adjusted by Bonferroni correction.

#### Inference of causal network structure and identification of key causal regulators

In order to establish a causal relationship or dependency between nodes in the network, we constructed causal structures from the gene expression data. A popular method for constructing causal structures is probabilistic graphical models, which are used to analyze and visualize the conditional independence relationships between variables^25^. The structure of conditional independence among variables is usually represented as a directed acyclic graph (DAG), where each node represents a variable (expression of a gene) and each directed edge represents a direct cause. The PC algorithm (PC stands for the initials of its inventors Peter Spirtes and Clark Glymour) was used to learn causal structure in R package pcalg^25^, setting the significant level of the conditional independence test (partial correlation) α = 0.01. The PC algorithm starts from a complete undirected graph and deletes recursively edges based on conditional independence decisions. This yields an undirected graph that can then be partially directed. With high-dimensional datasets when the distribution of variables is multivariate normal, the partial correlation test is the most efficient tool to implement the conditional independence test in the PC algorithm^26^.

We intersected the full causal network with the Yellow module to identify the causal regulators (i.e., key drivers) that primarily regulated the expression of the module through key driver analysis (KDA). KDA was developed to identify key causal regulators for a particular gene set of interest with respect to a given regulatory network and recently has been successfully applied to identify local hubs as potential key drivers of pathological perturbations in disease-associated modules in multiple complex diseases^8, 27, 28^. Briefly, the KDA took as input a set of genes (**G**) and a gene causal (directed) network **N**. We calculated the size (*μ*) of the h-layer neighborhood (HLN) downstream and the out-degrees (*d*) for each node in the subnetwork resulting from the intersection between causal network and the Yellow module. Genes with the size of their HLN greater than $\overline{\mu }+\sigma (\mu )$ were nominated as causal regulators. The regulators with degree above $\overline{d}+2\sigma (d)$, become key causal regulators of a corresponding network module^27^.

#### Integrative analysis of miRNA and mRNA expression data

In order to assess the role of miRNA in regulating obesity-associated module genes in pathological processes, we integrated mRNA and miRNA expression profiles from the same 200 subjects using miRComb R package (http://mircomb.sourceforge.net). MiRNA abundance was quantified using next-generation sequencing. After quality control and normalization on the miRNA expression levels, detailed in the study by Civelek, *et al*.^13^, 356 miRNA species were reliably quantified that were expressed in human adipose tissue. The log2 of the normalized expression values for the 356 miRNAs were used for subsequent analysis.

Firstly, miRComb package calculated Pearson correlation of the expressions of all possible combinations of significantly differentially expressed mRNAs and miRNAs. Secondly, miRNA-mRNA pairs were identified by screening databases for known and predicted miRNA-mRNA target pairs in the MicroCosm database^29^. The package assigned a p-value to each miRNA-mRNA pair according to the MicroCosm database (p_database) which predicted mRNA-miRNA interactions based on miRNA and mRNA 3′UTR alignment sequences^29^. Finally, only those pairs with significant negative correlation and predicted by the MicroCosm database as potential targets were selected as potential functional miRNA-mRNA interactions.

### Replication Stage (II)

We studied module preservation and gene association with obesity in other four test gene expression profiles in adipose tissue from obese (BMI > 30) and non-obese subjects. Gene expression data sets were downloaded from GEO (accession number: GSE25401, GSE64567), and from ArrayExpress (accession number: E-MTAB-54), which contained whole-genome expression profiling on abdominal (ABD) and gluteal (GLU) adipose tissue. Each data set was subjected to stringent quality control. Potential outliers were identified by clustering and PCA and then removed.

#### Module preservation

Network module preservation statistics assess whether modules identified in the discovery network remain connected in the test network (density), and whether node connections are similar between the discovery and the test network (connectivity). To assess the preservation of modules discovered from GSE32512 in other four test expression data sets, we used the network module preservation statistics implemented in the function *modulePreservation* (100 permutations) in the WGCNA R package^30, 31^. Module preservation statistics were calculated without the need to define modules in the test dataset.

Although there are many statistics to determine which module properties are preserved, we focused on the *Zsummary* statistic which summarizes evidence that a module is preserved more significantly than a random sample of all network genes^30^. The following thresholds were suggested by simulation results in Langfelder *et al*.’s study^30^: *Zsummary* < 2 implies no evidence for module preservation, 2 < *Zsummary *< 10 implies weak to moderate evidence, and *Zsummary *≥ 10 implies strong evidence for module preservation.

#### Meta-analysis of module genes

In GSE32512 and other four test gene expression profiles, the associations between genes of the Yellow module and obesity were tested by a linear model adjusting for age using LIMMA package^17^. For each coefficient in the linear model, empirical Bayes moderated *t*-statistics and their associated p-values were calculated to assess the significance of the observed expression changes^17^. P-values were adjusted for multiple testing by controlling for false discovery rate (FDR) using the Benjamini-Hochberg (BH) method^32^. In order to identify differentially expressed genes (DEGs) associated with obesity consistently across these five gene expression datasets, two meta-analysis methods were applied. First, a nonparametric meta-analysis method, Fisher’s method was performed to combine p-values from individual data sets to identify DEGs with large effect sizes across these five gene expression datasets. Fisher’s method was used to combine *p*-values by LIMMA from individual data sets. The combined Fisher’s statistic ${\chi }^{2}=-2\sum _{i=1}^{k}\mathrm{ln}({p}_{i})$ followed a *χ* ^2^ distribution with 2k degrees of freedom (where k is the number of datasets) under the null hypothesis. Note that smaller *p*-values contributed larger scores to the Fisher’s *χ* ^2^ statistic. Second, because Fisher’s method could not distinguish direction of effect sizes, a second meta-analysis method was used to combine effect sizes across all datasets into a meta-effect size to estimate the magnitude of gene expression change. Cochran’s Q statistic and *I* ^*2*^ were calculated as measures of between-study heterogeneity for each gene. Genes with Q test’s p-value < 0.05 or *I* ^2^ > 50% were assessed by a random effects model that allows heterogeneity in the effect sizes between different datasets^33^; the remaining genes were assessed by a fixed effects model, which assumed that the standardized effect sizes can be combined across different data sets and that the variations in observed effects were due only to random error^34^. Differences in each gene’s expression between obese and normal groups were expressed as the standardized mean difference (SMD). The z-statistic for each gene was computed as a ratio of the pooled SMD to its standard error, and the result was compared with 1000 permutations to obtain a nominal p-value using R *MetaDE* package^35^. P-values were corrected for multiple testing using the BH method.

### Validation Stage (III)

We utilized five experiments including four longitudinal dietary intervention studies in human abdominal subcutaneous adipose tissue to assess supportive biological information for future functional validation of hub genes’ involvement in adipose tissue metabolism. These experiments included the gene expression data in adipose tissue from obese or overweight human subjects in The Preventing Overweight Using Novel Dietary Strategies (POUNDS LOST) Trial (experiment 1)^36^, gene expression profile in adipose tissue from obese human subjects during weight loss and weight maintenance induced by LCD (experiment 2)^37^, real-time quantitative PCR (RT-qPCR) analysis of the effects of a longitudinal dietary intervention on human adipose tissue in the Diet, Obesity and Genes (Diogenes) intervention trial (experiment 3)^38, 39^, gene expression profile of metabolic responses and changes in adipose tissue from lean and overweight subjects during HFD overfeeding (experiment 4)^40^ and gene expression profile *in vitro* differentiated adipocytes from healthy subjects (experiment 5)^41^.

#### Experiment 1: Differential gene expression in adipose tissue from obese or overweight human subjects in the POUNDS LOST trial

The POUNDS LOST Trial (clinical trial reg. no. NCT00072995) is a 2-year randomized intervention trial which tested the effect of energy-reduced diets on weight loss in overweight or obese subjects^36^. We examined the differential gene expression of hub genes in human adipose tissue between baseline and 6-month weight loss intervention.

The POUNDS LOST Trial was conducted at Boston, MA, and Baton Rouge, LA, in 2004–2007. The study was approved by the human subjects committee at the Harvard School of Public Health and Brigham and Women’s Hospital, Boston, MA; the Pennington Biomedical Research Center of the Louisiana State University, Baton Rouge, LA; and a data and safety monitoring board appointed by the National Heart, Lung, and Blood Institute. All participants gave written informed consent. Detailed information on the The POUNDS LOST Trial study design and methods was previously described^36^. Major criteria for study exclusion were the presence of diabetes or unstable cardiovascular disease, the use of medications that affect body weight, and insufficient motivation.

A total of 811 overweight or obese subjects (BMI ≥ 25 and ≤ 40 kg/m^2^), age 30–70 years, were randomly assigned to one of weight loss diets. Each participant’s caloric prescription represented a deficit of 750 kcal per day from baseline, as calculated from the person’s resting energy expenditure and activity level. After an overnight fast, volunteers were biopsied by using Bergstrom technique with suction (Micrins Inc., Lake Forest, IL, USA) from abdominal subcutaneous adipose tissue (~500 mg). Total RNA was extracted by Trizol (Invitrogen, Carlsbad, CA, USA). The quantity and integrity of the RNA was analyzed by spectrophotometry and gel electrophoresis. Gene expression was measured by direct hypbridization using the Illumina HT-12 v3 expression beadchip (Illumina, San Diego, CA, USA). Gene expression profiles were assessed at baseline and at six months from 102 participants. After background correction and normalization, 13,948 of 48,803 probes had detection p-value < 0.01 (unpublished data). The differential expression of hub genes in the Yellow module between baseline and after 6 months intervention was conducted in LIMMA package and p-values were corrected for multiple testing using the BH method.

#### Experiment 2: Differential gene expression in adipose tissue from obese human subjects during weight loss and weight maintenance

To examine the differential gene expression of hub genes in human adipose tissue during diet-induced weight loss and weight maintenance after weight loss, we downloaded and analyzed the publicly available data set from GEO (accession number: GSE35411)^37^. RNA from abdominal subcutaneous adipose tissue from nine obese subjects was obtained and analyzed at baseline, after weight reduction on a low calorie diet (LCD), and after a period of group therapy in order to maintain weight stability. RNA profiling was performed using Affymetrix Human HG U133 Plus 2.0 arrays. Expression values were normalized by RMA algorithms^42^, implemented in R within the Affy package^43^. Differential expression analysis across three time points was conducted in LIMMA package. We focused on differences at the later time points by investigating contrasts of differences between each time point. LIMMA gave a moderated *F* statistic which can be used to test whether all contrasts were zero simultaneously, that is, whether there was no difference between the three time points. P-values were corrected for multiple testing using the BH method.

#### Experiment 3: Real-time quantitative PCR analysis of the effects of a longitudinal dietary intervention on human adipose tissue in Diogenes intervention trial

In order to decipher the adipose tissue response to diet-induced weight changes, another study focused on 221 genes during a longitudinal dietary intervention, which was part of the Diogenes intervention trial. A detailed description of the trial can be found in previous publications^38, 39^. Abdominal subcutaneous adipose tissue was obtained from 135 obese individuals at baseline, after a weight loss phase of 8-week LCD and after an *ad libitum* 26-week weight maintenance diet (WMD). After RNA extraction the mRNA levels of a panel of 221 genes selected from previous published and unpublished DNA microarray analyses was assessed using high throughput real-time quantitative PCR^39^. The processed RT-qPCR data files were downloaded from GEO (accession number: GSE60946). The differential expression analysis and multiple testing were the same with GSE35411 (Experiment 2).

#### Experiment 4: Differential expression in adipose tissue development and growth during overfeeding in human subjects

To further characterize the metabolic responses and changes of hub genes in gene expression that take place in adipose tissue during the early adaptive response to weight gain, we downloaded and analyzed the publicly available data set from GEO (accession number: GSE28005)^40^. In total, 13 subjects (7 overweight and 6 lean subjects) were involved in a high fat diet (HFD) for 56 days. Adipose tissue biopsies were taken at Day 0, Day 14 and Day 56. RNA profiling in adipose tissue was performed using Affymetrix Human HG U133 Plus 2.0 arrays. Expression values were normalized by Robust Multi-array Analysis (RMA) algorithms^42^, implemented by the *Affy* package in R^43^. Statistical analysis was also performed in LIMMA package. There were two groups (overweight and lean) and each subject was measured at Day 0, Day 14 and Day 56. Determining the differentially expressed genes meant studying contrasts of the various group × time levels. We were mainly interested in differences between the overweight and lean group at each time point. So a possible set of contrasts to investigate was “overweight0-lean0”, “overweight14-lean14”, “overweight56-lean56”. Similarly, a moderated *F* statistic was used to test whether all contrasts were zero simultaneously, that is, whether there was no difference between the two groups at any of the three time points. P-values were corrected for multiple testing using the BH method.

#### Experiment 5: Differential expression during differentiation of primary human adipocytes

To determine whether the hub genes were differentially expressed during *in vitro* differentiation of primary human adipocytes, we analyzed gene expression profiles of subcutaneous adipose tissue obtained from healthy subjects undergoing cosmetic liposuction (n = 12)^41^. The gene expression dataset was publicly accessible via GEO (accession number: GSE25910). Preadipocytes were isolated from the adipose tissue and in *vitro* differentiated to adipocytes. The cells were lysed at day 4/5 (early), 8 (middle) and 12 (late) of differentiation. From the samples, biotinylated complementary RNA was prepared and hybridized to Affymetrix GeneChip Human Gene 1.0 ST Array (Affymetrix Inc., Santa Clara, CA). Pre-processing and quality control was performed using the Affymetrix Expression Console version 1.1 by summarization, background correction and normalization^41^. Differential expression analysis across three time points was the same with GSE35411 (Experiment 2).

## Results

Our systems genetics framework for underlying molecular mechanisms of obesity was shown in Fig. 1. There were three stages in the framework. For each gene expression dataset in the present study, the detailed characteristics of samples, type of gene chip, tissue and number of outliers were shown in Supplementary Table S1.

### Construction of coexpression networks and identification of obesity-associated gene coexpression modules

**Figure 2:**
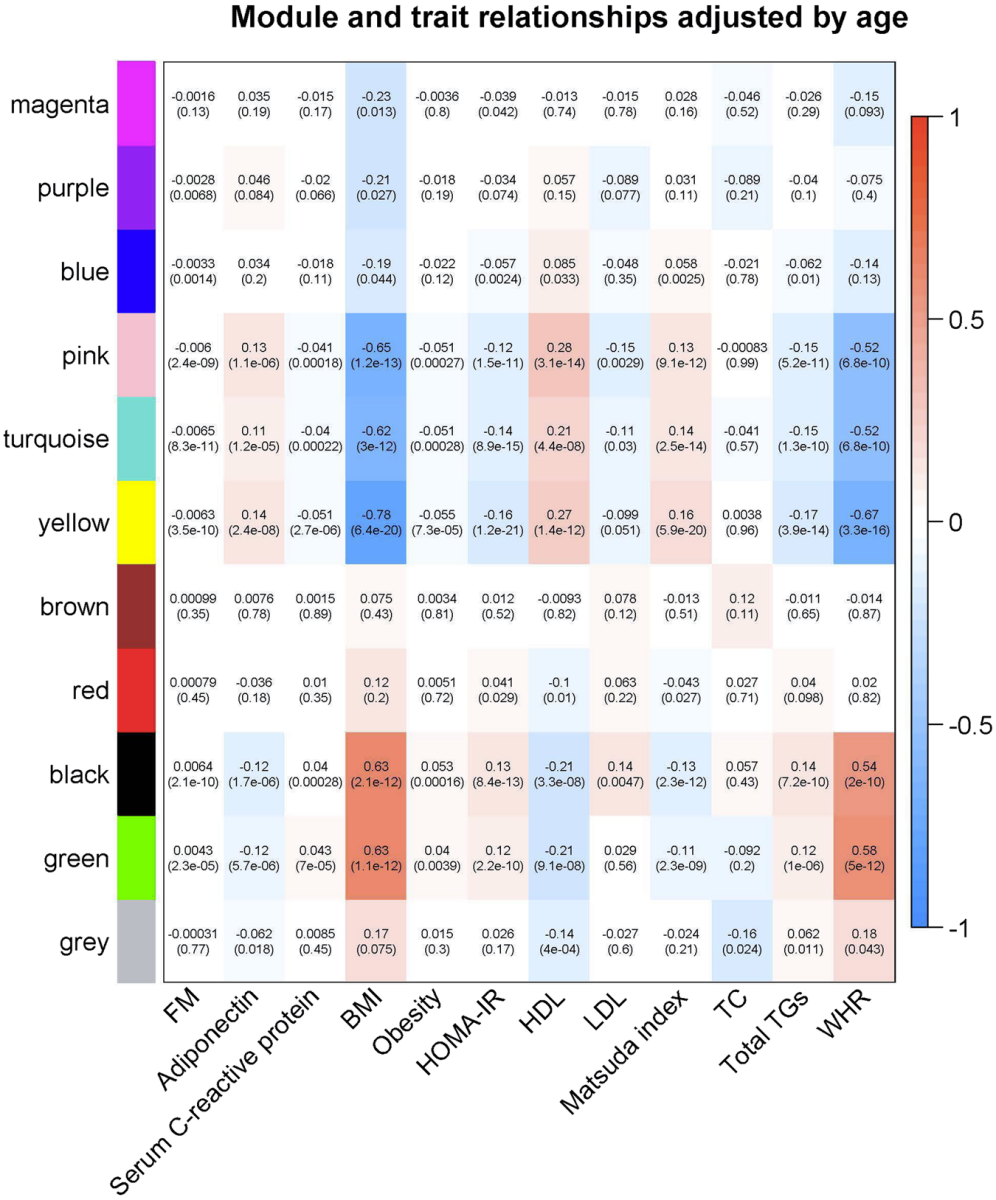
Module-trait relationships adjusted by age. Each row corresponded to a module eigengene, and each column to a trait. Numbers in the table report the age-adjusted correlations of the corresponding module eigengenes and traits, with the p-values printed below the correlations in parentheses. The table was color coded by correlation according to the color legend.

In total, 3035 genes, which were nominally significant GWAS genes with gene-wide p-value ≤ 0.05 for BMI and measured in GSE32512, were used to construct the coexpression networks in WGCNA package. Shown in Supplementary Fig. S1, 11 modules were identified by WGCNA and module sizes ranged from 41 (Purple module) to 492 genes (Turquoise module). Five modules (Pink, Turquoise, Yellow, Black, and Green) were significantly associated with both obesity and BMI at FDR < 0.001. Especially, the Yellow module (module size = 119 genes) showed the most significant result (p-value = 6.4E-20 for BMI and p-value = 7.3E-05 for obesity). We observed that ME of the Yellow module explained 7.0% of obesity variation and 34.2% of BMI variation, the latter of which was the largest among all the modules and relatively high considering BMI was a complex trait. We also observed that FM, CRP, TG, WHR, and HOMA-IR were significantly negatively correlated with the Yellow module, while adiponectin, HDL and Matsuda index were significantly positively correlated with the Yellow module (Fig. 2). Similarly, the Yellow module ME accounted for 17.5% of the variation of FM, 36.8% of HOMA-IR, 14.0% of adiponectin, 22.0% of HDL, 34.3% of Matsuda index, 24.8% of TG and 28.3% of WHR.

Based on the biological significance, the Yellow module appeared to play an important biological role in metabolism. We found that there was a significant positive correlation between module membership (MM) and Gene significance (GS) in the Yellow module (*r* = 0.62, p-value = 5.5E-14) (Supplementary Fig. S2). In total, there were eight hub genes (*GPD1L*, *CCDC50*, *NAALAD2*, *ALDH1L*, *ADH1B*, *ADH1A*, *PCCA* and *ORMDL3*) identified by the criteria in the Yellow module. Out of 97 loci identified with genome-wide significance by GIANT^4^, 32 genes were measured in GSE32512 and 18 of them failed to fit within a distinct group and were assigned to the “grey” module. Only one gene *EPB41L4B* was contained in the Yellow module. Although *EPB41L4B* was not identified as a hub gene (MM = 0.67), it reached the genome-wide significance and therefore was included into the subsequent analyses together with the hub genes.

Except for LDL and TC, all other traits had significant gene expression findings at FDR < 0.05 (Supplementary Table S2). We then conducted GO enrichment analysis for gene content of the Yellow module using the WebGestalt website (http://genereg.ornl.gov/webgestalt)^24^. The Yellow module genes showed six significantly enriched GO terms at Bonferroni-corrected p-value < 0.05 (Supplementary Table S3), which included cytoplasm, protein complex binding, cytoplasmic part, intracellular part, pore complex and alcohol dehydrogenase activity, zinc-dependent. Interestingly, *ADH1A* and *ADH1B*, two hub genes in the Yellow module, were enriched for GO term alcohol dehydrogenase activity, zinc-dependent (Fold enrichment = 71.42, Bonferroni-correct p-value = 4.77E-02).

The reported endogenous gene expression of hub genes during mouse development from MGI web tool was shown in Supplementary Table S4. In particular, all hub genes were reported to express in nervous system. Especially, GPD1L was reported to associate with Brugada syndrome^44^. ADH1A and ADH1B were associated with alcohol dependence^45^.

### Inference of causal network structure and identification of key causal regulators

**Figure 3:**
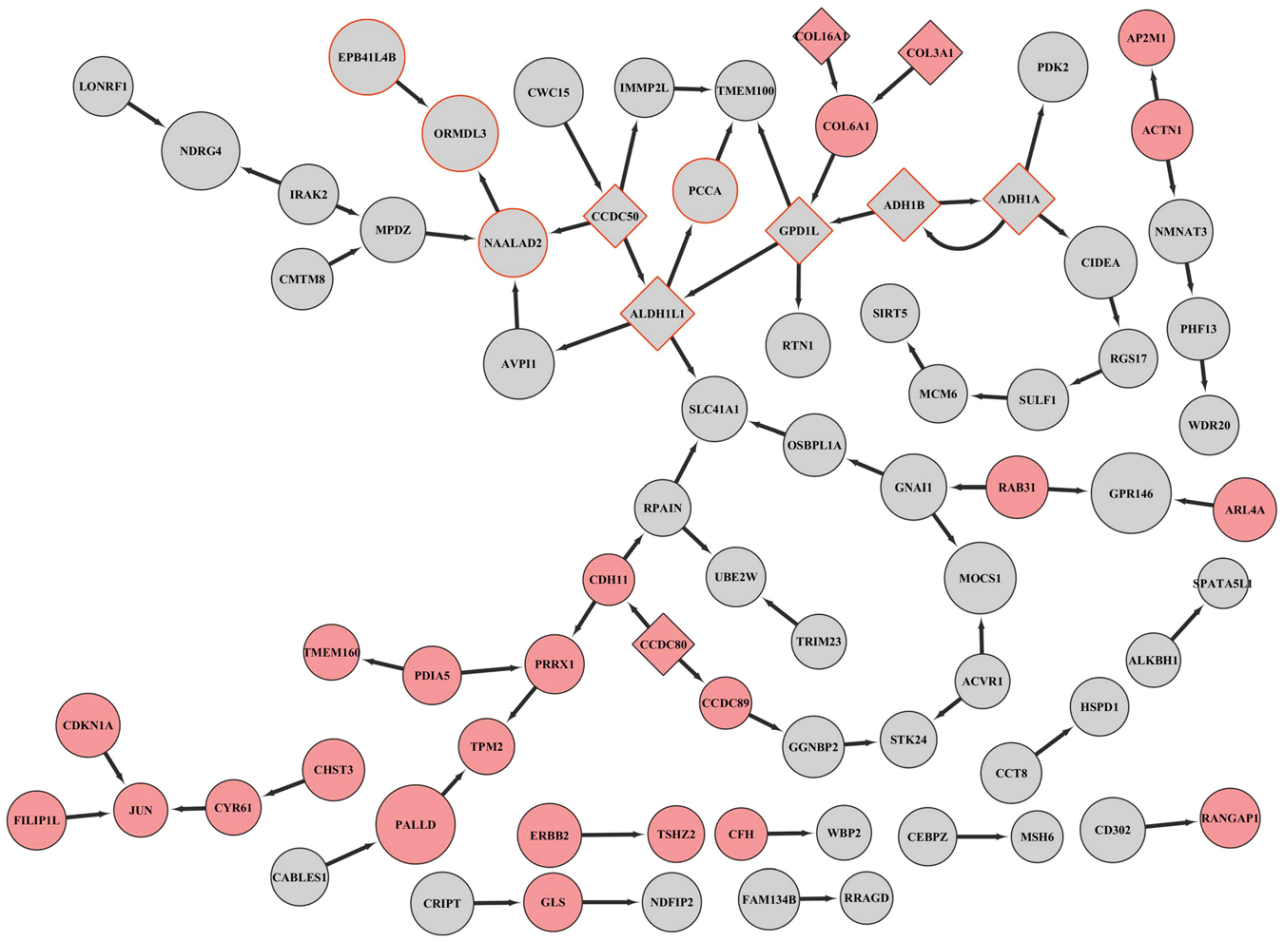
Causal network structure of the Yellow module. Each node represented a gene and each directed edge indicated a causal link between genes. Key driver genes were represented by diamond nodes. Node sizes were proportional to −log10 (GS p-value) of each gene. All hub genes’ borders were in red. Node color was shown in red and grey, where grey color was indicative of an upregulation in obese subjects, and red color an upregulation in non-obese subjects.

The causal structure based on the conditional independence test (partial correlation, detailed in Methods) contained 3843 significant causal relationships among 3035 genes in GSE32512. We intersected the causal network with the Yellow module to identify the key drivers which primarily regulated the expression of the module. 7 key drivers were identified including *ADH1A, ADH1B, ALDH1L1, GPD1L, CCDC50, CCDC80* and *COL16A1*, shown in Fig. 3. Not surprisingly, the first five key drivers were hub genes defined above by GS and MM, as KDA relied on precomputed gene-gene interaction networks and topology-based gene ranking. Especially, based on the significant level of the conditional independence test α = 0.01, the causal inference revealed the causal links among all eight hubs and the top GWAS loci *EPB41L4B* (Fig. 3).

### Integrative analysis of miRNA and mRNA expression data

For the 119 genes in the Yellow module, 35 out of 852 miRNA-mRNA interactions were significant (FDR < 0.05). The network of miRNA-mRNA interactions with FDR < 0.05 was shown in Supplementary Fig. S3. For the hub genes, shown in Table 1, four pairs were identified at FDR < 0.05, including *ALDH1L1* and hsa-miR-342-5p (*r = *−0.27, FDR = 1.76E-03), *NAALAD2* and hsa-miR-140-3p (*r = *−0.29, FDR = 7.98E-04), *ORMDL3* and hsa-miR-362-3p (*r = *−0.20, FDR = 3.23E-02), and *PCCA* and hsa-miR-301b (*r = *−0.25, FDR = 5.42E-03). The hub gene *GPD1L* was negatively correlated with hsa-miR-210 (*r* = −0.17, FDR = 7.91E-02), which was experimentally validated and confirmed that the predicted hsa-miR-210 binding site on *GPD1L* 3′ UTR is a *bona fide* target and that has-miR-210 regulated *GPD1L* protein levels through direct interaction with the mRNA 3′ UTR^46^. There was no predicted miRNA-mRNA target pair in the MicroCosm database for gene *ADH1B*.

**Table 1:** Hub genes in the Yellow module.

Gene	Gene-level p-value^a^	GS	GS p-value	MM	MM p-value	Rank^b^	miRNA	*r* ^c^	p-value	FDR
GPD1L	2.67E-02	−0.23	1.06E-03	0.90	1.29E-71	1	hsa-miR-210	−0.17	7.37E-03	7.91E-02
CCDC50	2.12E-02	−0.20	4.68E-03	0.85	6.79E-58	2	hsa-miR-501-3p	−0.19	3.93E-03	5.05E-02
NAALAD2	8.05E-03	−0.24	5.76E-04	0.85	1.47E-57	3	hsa-miR-140-3p	−0.29	1.97E-05	7.98E-04
ALDH1L1	4.94E-03	−0.28	7.13E-05	0.85	3.15E-57	4	hsa-miR-342-5p	−0.27	5.13E-05	1.76E-03
ADH1B	3.14E-02	−0.27	8.82E-05	0.85	4.39E-56	5	—	—	—	—
ADH1A	5.00E-02	−0.26	1.83E-04	0.82	2.34E-50	6	hsa-miR-590-3p	−0.09	1.06E-01	4.46E-01
PCCA	2.38E-02	−0.21	2.68E-03	0.82	3.87E-49	8	hsa-miR-301b	−0.25	2.08E-04	5.42E-03
ORMDL3	3.87E-03	−0.29	3.04E-05	0.80	3.29E-45	9	hsa-miR-362-3p	−0.20	2.12E-03	3.23E-02
EPB41L4B^d^	7.42E-05	−0.29	3.47E-05	0.67	2.63E-27	28	has-miR-3613-3p	−0.11	6.46E-02	3.4E-01

GS, gene significance; MM, module membership. ^a^Gene-level p-value generated by ProxyGeneLD in the GIANT GWAS. ^b^Rank based on MM. ^c^Correlation betwwen miRNA and mRNA. For gene ADH1B, there was no predicted miRNA-mRNA target pair in the MicroCosm database. ^d^One of 97 BMI loci identified by GIANT.

### Preservation of Yellow module in other four datasets

The Yellow module showed strong evidence for preservation in three datasets, including E-MTAB-54 ABD, GLU and GSE25401, with *Zsummary* = 14, 10 and 14, respectively. However, in GSE64567, the Yellow module showed no evidence for module preservation (*Zsummary* = 1.6).

### Meta-analysis of module genes

In the meta-analysis of combining p-values across individual gene expression profiles using Fisher’s method, 73 genes in the Yellow module showed significant results with obesity (FDR < 0.05) (Supplementary Table S5). The second meta-analysis approach combined effect sizes across individual gene expression profiles, resulting in the identification of genes that were upregulated and downregulated. 84 genes in the Yellow module were identified to differentially express between obese and non-obese subjects with FDR < 0.05 (Supplementary Table S6). Specifically, 66 overlapped genes showed significant results in both meta-analysis methods. The results of meta-analysis for the hub genes were showed in Table 2. All hub genes and gene *EPB41L4B* had negative SMDs with 95% CIs not including 0, which indicated that they were significantly downregulated in obese subjects.

**Table 2:** The results of meta-analysis for hub genes in the Yellow module.

Gene	Meta-analysis (Fisher)			Meta-analysis (Effect size)		
	Fisher	p-value	FDR	SMD (95%CI)	p-value	FDR
GPD1L	75.85	3.34E-13	2.47E-09	−1.29 (−1.78, −0.80)	1.13E-06	3.63E-05
CCDC50	37.02	1.14E-05	4.58E-04	−0.73 (−0.97, −0.48)	5.17E-09	2.89E-07
NAALAD2	94.58	6.66E-16	3.70E-13	−1.11 (−1.50, −0.72)	2.18E-08	8.45E-07
ALDH1L1	46.19	2.19E-07	2.38E-05	−0.81 (−1.06, −0.56)	9.68E-11	1.21E-08
ADH1B	61.55	2.31E-10	1.42E-07	−0.88 (−1.13, −0.63)	2.94E-12	7.24E-10
ADH1A	52.2	1.54E-08	3.45E-06	−0.77 (−1.02, −0.53)	5.97E-10	5.07E-08
PCCA	71.75	2.03E-11	1.74E-09	−0.86 (−1.09, −0.63)	9.15E-14	8.24E-12
ORMDL3	105.5	4.30E-18	4.51E-15	−1.04 (−1.55, −0.54)	1.18E-05	1.63E-04
EPB41L4B^a^	41.80	1.48E-06	1.05E-04	−0.65 (−0.90, −0.41)	1.29E-07	4.17E-06

SMD, standardized mean difference. ^a^One of 97 BMI loci identified by GIANT.

### Expression profiling experiments in human adipose tissue

In experiment 1 (The POUND LOST Trial)^36^, we investigated the effects of a 6-month weight-loss diet intervention on hub genes expression in 102 obese or overweight subjects. Although all the hub genes were upregulated during the 6-month weight loss (Fold change >1), five hub genes (*GPD1L, ADH1B, ADH1A, ORMDL3* and *EPB41L4B*) reached the statistical significance with FDR < 0.05 (Table 3).

**Table 3:** The results of five experiments for hub genes in the Yellow module.

Gene	Experiment 1 (The POUNDS LOST Trial)			Experiment 2 (GSE35411)			Experiment 3 (GSE60946)			Experiment 4 (GSE28005)			Experiment 5 (GSE25910)		
	FC^a^	p-value	FDR	F	p-value	FDR	F	p-value	FDR	F	p-value	FDR	F	p-value	FDR
GPD1L	1.4	5.69E-06	1.57E-03	8.82	1.17E-03	1.95E-02	3.64	2.73E-02	3.27E-02	17.22	4.57E-07	2.01E-05	4.13	2.39E-02	3.88E-02
CCDC50	1.08	2.48E-01	3.01E-01	1.73	1.97E-01	3.84E-01	—	—	—	12.13	1.29E-05	2.16E-04	15.34	1.35E-05	4.94E-05
NAALAD2	1.16	1.61E-01	2.37E-01	5.51	1.00E-02	6.81E-02	—	—	—	18.94	1.68E-07	1.20E-05	9.98	3.33E-04	8.85E-04
ALDH1L1	1.21	2.79E-01	3.20E-01	2.1	1.42E-01	3.21E-01	—	—	—	18.91	1.71E-07	1.20E-05	155.41	6.75E-19	7.90E-17
ADH1B	1.5	8.23E-04	1.49E-02	17.86	1.25E-05	2.29E-03	—	—	—	15.83	1.08E-06	3.83E-05	2.02	1.47E-01	1.85E-01
ADH1A	1.54	3.75E-05	3.24E-03	7.82	2.16E-03	3.05E-02	—	—	—	5.92	2.21E-03	1.03E-02	2.72	7.86E-02	1.11E-01
PCCA	1.07	2.05E-01	2.71E-01	7.86	2.10E-03	3.05E-02	9.45	9.79E-05	1.92E-04	4.41	9.80E-03	3.10E-02	11.92	9.80E-05	2.94E-04
ORMDL3	1.29	3.07E-03	2.82E-02	4.51	2.07E-02	1.05E-01	—	—	—	23.27	1.69E-08	3.26E-06	78.53	3.80E-14	1.11E-12
EPB41L4B^b^	1.19	3.34E-03	2.93E-02	3.97	3.11E-02	1.42E-02	—	—	—	15.38	1.43E-06	3.92E-05	20.55	9.34E-07	4.55E-06

“—” Gene was not measured by the RT-qPCR in the GSE60946. ^a^FC, Fold change (Weight loss vs. Baseline). ^b^One of 97 BMI loci identified by GIANT.

In experiment 2 (GSE35411)^37^, we examined the differential gene expression of hub genes in adipose tissue from nine obese subjects during diet-induced weight loss and weight maintenance after weight loss. After multiple testing correction, *GPD1L*, *ADH1B*, *ADH1A* and *PCCA* showed significant differential expression across the time course of baseline, weight-loss and weight maintenance (FDR < 0.05) (Table 3). Experiment 3 (GSE60946) had a study design similar to experiment 2. The sample size of experiment 3 was 135^38, 39^, much larger than experiment 2, although it used RT-qPCR to quantify mRNA levels of only 221 genes, in which two hub genes (*GPD1L* and *PCCA*) in the Yellow module were measured. Interestingly, hub gene *GPD1L* and *PCCA* both showed significant differential expression over three time points (baseline, weight-loss and weight maintenance), with FDR = 3.27E-02 and 1.92E-4, respectively (Table 3).

In experiment 4 (GSE28005)^40^, we examined changes of hub genes in gene expression of adipose tissue during the early adaptive response to weight gain induced by overfeeding. We observed that the expression of all the hub genes were significantly different between lean and overweight subjects over three time points (Day 0, 14 and 56), even after correction for multiple testing (FDR < 0.05) (Table 3).

In experiment 5 (GSE25910)^41^, we characterized the gene expression patterns during *in vitro* differentiation of primary human adipocytes. Apart from *ADH1A* and *ADH1B*, the other six hub genes and gene *EPB41L4B* were significantly differentially expressed across a time course of early, middle and late of adipocyte development (FDR < 0.05). Especially, gene *ALDH1L1* showed the most significant result even after multiple testing (FDR = 7.90E-17) (Table 3).

### The most promising candidate gene for obesity in adipose tissue

**Figure 4:**
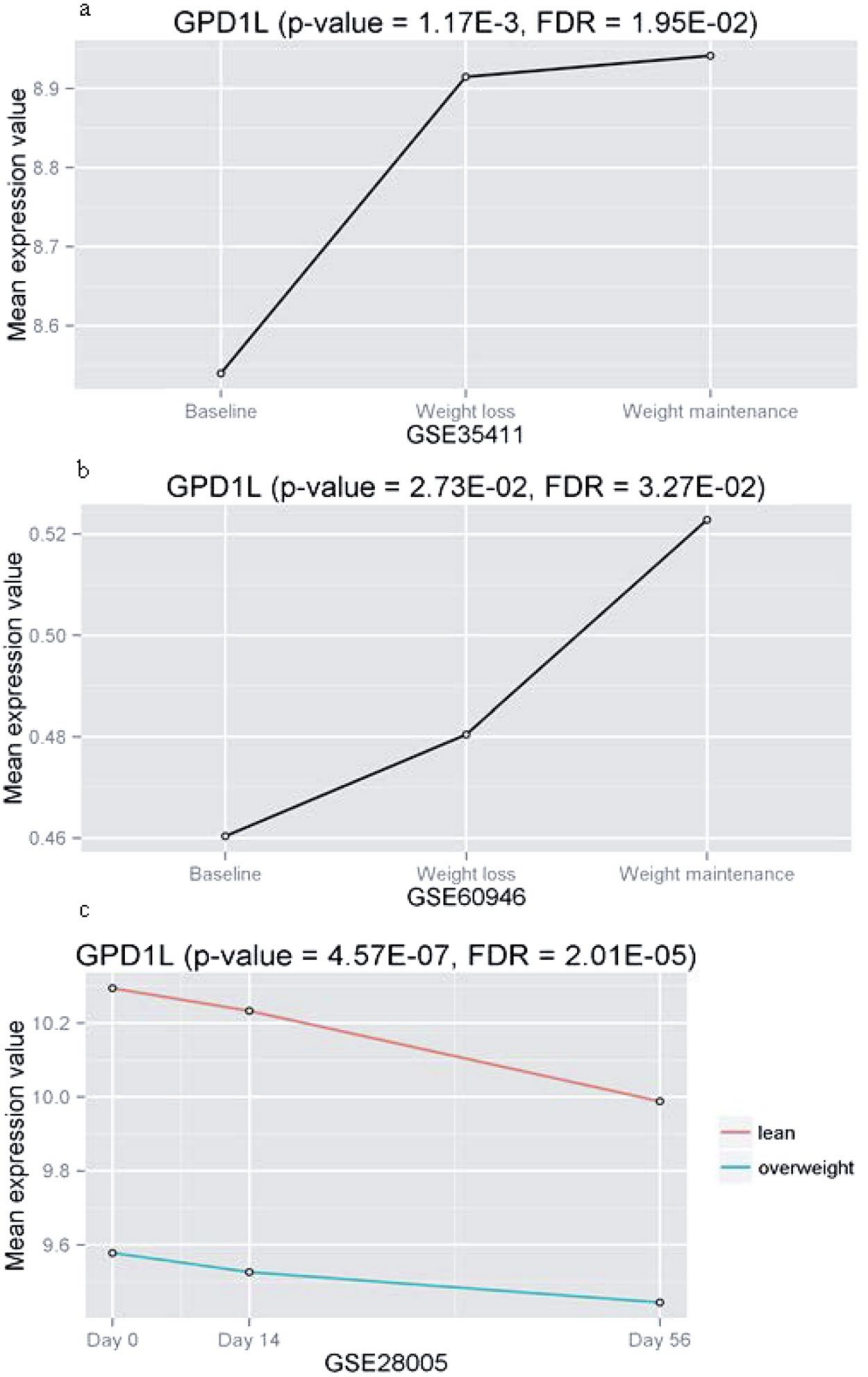
The most promising candidate gene for obesity, *GPD1L*. (**a** and **b**) The differential gene expression patterns in adipose tissue from obese human subjects during weight loss and weight maintenance in experiment 2 (GSE35411) and experiment 3 (GSE60946), respectively. (**c**) The differential expression pattern between overweight and lean subjects over day 0, 14 and 56. (experiment 4, GSE28005).

We sought to characterize hub genes as a way to prioritize and identify the most promising and potential causal genes for obesity. Gene *GPD1L* had the highest connectivity in the Yellow module and was identified a key causal regulator in the Yellow module. It was significantly negative association with BMI and obesity. And *GPD1L* expression was negatively correlated with miR-210, which was experimentally validated and confirmed to regulate *GPD1L* levels through direct interaction with the mRNA 3′ UTR. In the meta-analysis, *GPD1L* was identified to be significantly associated with obesity by Fisher’s method, as well as by combining effect size method (Supplementary Fig. S4). With age adjusted, *GPD1L* was also significantly positively associated with adiponectin, HDL and Matsuda index, negatively associated with FM, CRP, HOMA-IR, total TGs and WHR (Supplementary Table S2). Especially, the highly significant negative correlation of *GPD1L* with WHR and FM provided evidence for a potential role in central obesity which was highly related to insulin resistance. The negative association of *GPD1L* with the measure of insulin resistance HOMA-IR (β = −0.83, FDR = 1.99E-14) was shown in Supplementary Fig. S5. Moreover, *GPD1L* was upregulated in response to weight loss in the POUND LOST trial (experiment 1)^36^. Interestingly, in both experiments 2 and 3, *GPD1L* also showed a similarly upregulated expression pattern from baseline to weight maintenance induced by LCD (Fig. 4a and b)^37–39^. In experiment 4 (GSE28005), on the contrary, *GPD1L* was downregulated in response to weight gain induced by HFD overfeeding in lean and overweight subjects (Fig. 4c)^40^. In experiment 5 (GSE25910)^41^, *GPD1L* had significantly differential expression across a time course of early, middle and late of adipocyte development. Taken together, these findings in various metabolic measures provided strong supporting evidence for an important role for *GPD1L* in obesity.

## Discussion

Our systems genetics approach not only identified a Yellow module most significantly associated with obesity and other metabolic traits, but also identified eight hub genes and five of them were key causal regulators in the module. We identified miRNAs that significantly negatively correlated with the expression of hub genes, providing an understanding of the role of miRNA in regulating hub genes in pathological processes. A comprehensive characterization of module connectivity and hub genes’ association with obesity provided with critical insights into the underlying mechanisms. This novel approach resulted in the discovery of *GPD1L* as the most important candidate causal gene for obesity that may serve as an effective target for therapeutic intervention.

Gene *GPD1L* on chromosome 3p22.3 encodes the protein glycerol 3-phosphate dehydrogenase 1-like (*GPD1L*), which catalyzes the conversion of sn-glycerol 3-phosphate to glycerone phosphate. The encoded protein was found in the cytoplasm, associated with the plasma membrane^47^. Although the biological function of the gene *GPD1L* in human adipose tissue and obesity requires further investigation, our findings and other evidence supported that *GPD1L* in adipose tissue may play a pivotal role in the molecular mechanism of obesity. First, several lines of evidence suggest that adipose tissue is poorly oxygenated in obese human subjects, even in the early course of HFD feeding and mouse models of obesity, indicating that adipose tissue expansion in response to chronic and excessive nutrient consumption increased oxygen consumption and created a state of relative hypoxia due to mitochondrial dysfunction^48, 49^. Second, in mammals the response to low oxygen conditions was mediated primarily by gene expression changes induced by the hypoxia-inducible factor (*HIF*) family of transcription factors. In addition, it has been reported that adipocyte-specific *HIF-1α* overexpression in mice led to more severe obesity with increased white adipose tissue mass^50^, and insulin resistance with increased adipose tissue inflammation^51^. Several studies showed that adipocyte-specific *HIF-1α* knockout mice exhibited reduced fat formation, protection from HFD-induced obesity and insulin resistance compared with similarly fed knockout mice and wild-type controls^49, 52, 53^. Although the mechanism of *HIF-1α* effects on adiposity and obesity requires further investigation, compounds that inhibit *HIF-1α* activity in adipose tissue might have a significant therapeutic potential in reducing obesity and insulin resistance^53^. Third, interestingly, Kelly *et al*. identified the enzyme *GPD1L* as a novel regulator of *HIF-1α* stability and a direct target of miR-210 in its 3′ UTR^46^. miR-210 was regarded as “master miRNA” of hypoxic response as it has been found to be upregulated by hypoxia in virtually all cell types tested to date^54^. Kelly showed a model of a hypoxia-induced feedback loop, which was subsequently found and confirmed by another study^55^. Overexpression or knockdown of *GPD1L* levels resulted in a decrease or increase in *HIF-1α* stability and transcriptional activity, respectively, by regulating proline hydroxylation of *HIF-1α* at proline 564^46^. As oxygen levels decreased (hypoxia), *HIF-1α* protein and transcriptional activity increased, triggering accumulation of miR-210. Increased miR-210 directly repressed *GPD1L* protein expression through binding to 3′UTR of *GPD1L* gene and further repressed prolyl hydroxylases (PHDs) activity, subsequently promoting *HIF-1α* protein expression^46^. And the overexpression of *HIF-1α* led to insulin resistance, adipose tissue inflammation and obesity.

Consistent with the above feedback loop activity, the present study showed that *GPD1L* was negatively correlated with miR-210 and consistently downregulated in obese subjects. This is possibly because in obese adipose tissue the increased stability of *HIF-1α* was caused by decreased *GPD1L* due to the accumulation of miR-210. More importantly, in the present study, *GPD1L* was found to be downregulated in response to weight gain induced by HFD. Based on the hypoxia-induced feedback loop, HFD induced a state of relative hypoxia, resulting in the accumulation of miR-210 and subsequently the decreased *GPD1L*. On the contrary, *GPD1L* was found to be upregulated in obese subjects in response to weight loss and maintenance induced by LCD, because during LCD the state of relative hypoxia was attenuated, resulting in the decreased miR-210 and subsequently the increased *GPD1L*. The increased *GPD1L* inhibited *HIF-1α* activity in adipose tissue, leading to reduction in obesity and insulin resistance. Consistently, in the present study *GPD1L* was found to be significantly negatively correlated with the measure of insulin resistance, HOMA-IR.

**Figure 5:**
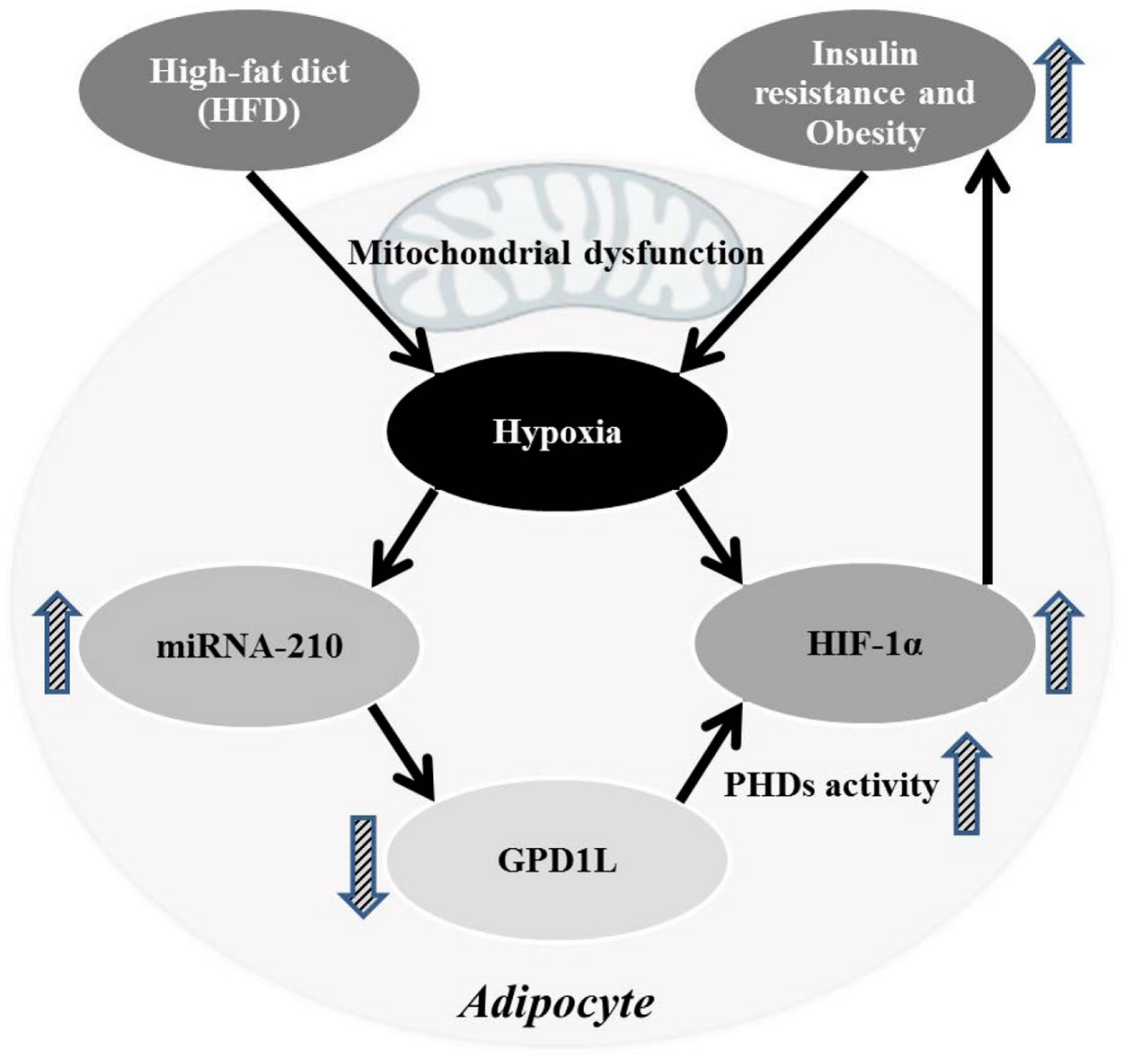
A potential mechanism of HFD-induced obesity based on hypoxia-induced feedback loop with miRNA-210 regulating *GPD1L*. During HFD, the oxygen levels decreased (a state of relative hypoxia), HIF-1α protein and transcriptional activity increased, triggering accumulation of miR-210. Increased miR-210 caused decreased *GPD1L* protein and a further inactivation of the PHDs, resulting in increased HIF-1α protein. Overexpression of HIF-1α developed insulin resistance with increased adipose tissue inflammation, resulting in fat accumulation and obesity. Obesity aggravated the hypoxia, resulting in a feedback regulatory loop.

Note that the associations of the Yellow module and key driver genes with obesity observed in the discovery stage alone did not support causal relationships, as the METSIM Study is cross-sectional. However, together with the findings from four clinical studies with longitudinal weight-loss and weight-gain interventions, we were able to infer causal links among diet interventions, miR-210, gene *GPD1L* and weight change in the mechanism of HFD-induced obesity (Fig. 5), which was worthy of further validation by functional experiments. The present study linked the regulation of miR-210 on *GPD1L*, the biological importance of *GPD1L* in adipose tissue, obesity and insulin resistance with the hypoxia-induced feedback loop. It indicated that increased *GPD1L* which can inhibit *HIF-1α* activity in adipose tissue might have significant therapeutic potential in reducing obesity and insulin resistance.

Two hub genes we identified, *ADH1A* and *ADH1B*, were also found to be candidate genes for obesity and insulin resistance by Winnier *et al*. in a study of human abdominal subcutaneous adipose tissue in 75 Mexican Americans^56^. Winnier’s study provided strong experimental support for the potential roles for *ADH1A* and *ADH1B* in obesity, insulin resistance and T2D. Both genes had almost identical tertiary structures and enzymatic mechanisms, which metabolize a wide variety of substrates, including ethanol, retinol, other aliphatic alcohols, hydroxysteroids, and lipid peroxidation products^57^. According to a microarray assay of 76 normal human tissues (http://BioGPS.org), *ADH1A* and *ADH1B* were most highly expressed in the liver and adipose tissue, respectively. One possible mechanism of *ADH1A* and *ADH1B* in adipose tissue and obesity has been proposed to explain their negative association with obesity-related traits and insulin resistance. They are involved in the reversible conversion of alcohol to fatty acids, subsequently producing acetyl-CoA via β-oxidation^56^. Therefore, elevated levels of *ADH1A* and *ADH1B* in human adipocytes could plausibly promote efficient metabolism of energy from alcohol, moderating its storage as fat^56^. Another hub gene *ALDH1L1*, which also plays an important role in ethanol metabolism, was found to be significantly downregulated in the obese compared with lean co-twins^58^. *ALDH1L1* appeared to be a chief regulator of cellular metabolism as it was strongly downregulated in certain physiological and pathological conditions, while its upregulation can produce drastic antiproliferative effects^59^.

Other hub genes, including *CCDC50*, *ORMDL3*, *PCCA* and *NAALAD2*, have diverse cellular functions and have not been previously implicated as obesity candidate genes. However, given their relationships with obesity and metabolic traits, and their expression patterns in the five experiments of validation stage, their functional characterization and specific roles in adipocyte metabolism in obesity are worthy of further investigation.

Although gnome-wide significant SNPs in GWAS explain only a small proportion of the heritability, known as “missing heritability”^60^, recent studies have shown that the total variance explained by all common variants has a large proportion of the heritability for complex traits^61^. Much of the missing heritability that could not pass the stringent genome-wide significance threshold can be found by using more advanced integrative methods. GWAS still may provide useful information about the putative causal relationship between genetic loci and traits, as genetic loci can impose disease risk via the products of the genome, such as gene expression, miRNA and protein level. Functional mechanisms of complex traits can be inferred when the risk loci are placed in a greater context, such as implicated tissues/cell-types and inter-connected networks, as gene functions are commonly dependent on their tissue context and the disordered interplay of tissue–specific processes^5^. Our system genetics approach demonstrated the power of discovering more prominent driver genes influencing complex traits and diseases by integrating risk genetic loci with gene expression profiling in disease-related tissues. Our results contextualized in human abdominal subcutaneous adipose tissue discrete BMI GWAS genes, which were functionally linked via gene regulatory networks important for obesity.

Gene *EPB41L4B* was identified as one of the top BMI loci by the GIANT study and we were able to follow up this gene as a Yellow module gene in our systems genetics framework. It was consistently downregulated in obese subjects and had a significantly differential pattern in adipose tissue during *in vitro* differentiation of primary human adipocytes. Besides, it was significantly downregulated and upregulated in response to weight gain and weight loss, respectively in the validation stage. The role of *EPB41L4B* gene in BMI regulation deserves further investigation.

Tissue and cell-type identity lie at the core of human physiology and disease etiology. It is very crucial to understand the genetic underpinnings of complex tissues and the role of genetic variants in disease specific tissues for disease etiology, in order to develop improved diagnostics and therapeutics^5^. Pathologically, obesity is mainly caused by dysfunctional adipose tissue^62^. Adipose tissue is one of the largest tissues in the human body and essential for normal energy homeostasis. Adipose tissue plays a key role in the control of energy balance by secreting e.g. hormones, cytokines and growth factors. During the time of excessive energy intake, insulin stimulates the uptake of glucose in fat, resulting in excessive accumulation of triacylglycerol in multiple fat depots. Therefore, adipose tissue may enlarge by increasing the size (hypertrophy) and number (hyperplasia) of adipocytes. Enlarged adipocytes lead to dysregulated secretion of adipokines and increased release of free fatty acids^14^. Dysfunction of normally insulin-sensitive adipose tissue has been repeatedly shown to be involved in important metabolic pathologies, such as insulin resistance, T2D, obesity and CVD^63^. It is suggested that adipose tissue can serve as a very important *in vivo* model to better understand complex molecular mechanisms underlying obesity^64, 65^. Therefore, all gene expression datasets in the present study were from adipose tissue.

In summary, our systems genetics approach revealed important candidate causal genes and mechanisms for obesity. Our approach showed the efficiency of mining and prioritizing GWAS results in publicly available gene expression profiling in disease-related tissues. More importantly, the most promising candidate causal gene *GPD1L* and its role in the etiology of HFD-induced obesity were highlighted. Our findings indicated that increased *GPD1L* which can inhibit *HIF-1α* activity in adipose tissue might have a significant therapeutic potential in reducing obesity and insulin resistance. Our novel and comprehensive systems genetics study provided strongly supportive biological information for further functional validation of the mechanism underlying obesity.

## Electronic supplementary material

Supplementary Dataset

Supplementary info

## References

[1] Bener A, Zirie M, Al-Rikabi A (2005). Genetics, obesity, and environmental risk factors associated with type 2 diabetes. Croatian medical journal.

[2] Bell CG, Walley AJ, Froguel P (2005). The genetics of human obesity. Nature reviews. Genetics.

[3] Stunkard AJ, Foch TT, Hrubec Z (1986). A twin study of human obesity. Jama.

[4] Locke AE (2015). Genetic studies of body mass index yield new insights for obesity biology. Nature.

[5] Greene CS (2015). Understanding multicellular function and disease with human tissue-specific networks. Nature genetics.

[6] Zhang, B. & Horvath, S. A general framework for weighted gene co-expression network analysis. *Statistical applications in genetics and molecular biology* **4**, Article 17, doi:10.2202/1544-6115.1128 (2005). 10.2202/1544-6115.1128 16646834

[7] Civelek M, Lusis AJ (2014). Systems genetics approaches to understand complex traits. Nature reviews. Genetics.

[8] Makinen VP (2014). Integrative genomics reveals novel molecular pathways and gene networks for coronary artery disease. PLoS genetics.

[9] Heneghan HM, Miller N, Kerin MJ (2010). Role of microRNAs in obesity and the metabolic syndrome. Obesity reviews: an official journal of the International Association for the Study of Obesity.

[10] Friedman Y, Balaga O, Linial M (2013). Working together: combinatorial regulation by microRNAs. Advances in experimental medicine and biology.

[11] Devlin B, Roeder K (1999). Genomic control for association studies. Biometrics.

[12] Hong MG, Pawitan Y, Magnusson PK, Prince JA (2009). Strategies and issues in the detection of pathway enrichment in genome-wide association studies. Human genetics.

[13] Civelek M (2013). Genetic regulation of human adipose microRNA expression and its consequences for metabolic traits. Human molecular genetics.

[14] Jung UJ, Choi MS (2014). Obesity and its metabolic complications: the role of adipokines and the relationship between obesity, inflammation, insulin resistance, dyslipidemia and nonalcoholic fatty liver disease. International journal of molecular sciences.

[15] Matthews DR (1985). Homeostasis model assessment: insulin resistance and beta-cell function from fasting plasma glucose and insulin concentrations in man. Diabetologia.

[16] Ritchie ME (2007). A comparison of background correction methods for two-colour microarrays. Bioinformatics.

[17] Smyth, G. K. Linear models and empirical bayes methods for assessing differential expression in microarray experiments. *Statistical applications in genetics and molecular biology* **3**, Article 3, doi:10.2202/1544-6115.1027 (2004). 10.2202/1544-6115.1027 16646809

[18] Troyanskaya O (2001). Missing value estimation methods for DNA microarrays. Bioinformatics.

[19] Hagg S (2015). Gene-based meta-analysis of genome-wide association studies implicates new loci involved in obesity. Human molecular genetics.

[20] Langfelder P, Zhang B, Horvath S (2008). Defining clusters from a hierarchical cluster tree: the Dynamic Tree Cut package for R. Bioinformatics.

[21] Ghazalpour A (2006). Integrating genetic and network analysis to characterize genes related to mouse weight. PLoS genetics.

[22] Gargalovic PS (2006). Identification of inflammatory gene modules based on variations of human endothelial cell responses to oxidized lipids. Proceedings of the National Academy of Sciences of the United States of America.

[23] Blake JA, Richardson JE, Bult CJ, Kadin JA, Eppig JT (2003). MGD: the Mouse Genome Database. Nucleic acids research.

[24] Zhang B, Kirov S, Snoddy J (2005). WebGestalt: an integrated system for exploring gene sets in various biological contexts. Nucleic acids research.

[25] Kalisch, M., MÃ¤chler, M., Colombo, D., Maathuis, M. H. & BÃ¼hlmann, P. Causal Inference Using Graphical Models with the R Package pcalg. *2012* **47**, 26, doi:10.18637/jss.v047.i11 (2012).

[26] Le TD (2013). Inferring microRNA-mRNA causal regulatory relationships from expression data. Bioinformatics.

[27] Zhang B (2013). Integrated systems approach identifies genetic nodes and networks in late-onset Alzheimer’s disease. Cell.

[28] Huan T (2015). Integrative network analysis reveals molecular mechanisms of blood pressure regulation. Molecular systems biology.

[29] Griffiths-Jones S, Saini HK, van Dongen S, Enright AJ (2008). miRBase: tools for microRNA genomics. Nucleic acids research.

[30] Langfelder P, Luo R, Oldham MC, Horvath S (2011). Is my network module preserved and reproducible?. PLoS computational biology.

[31] Langfelder P, Horvath S (2008). WGCNA: an R package for weighted correlation network analysis. BMC bioinformatics.

[32] Ferreira, J. A. The Benjamini-Hochberg method in the case of discrete test statistics. *The international journal of biostatistics* **3**, Article 11 (2007). 10.2202/1557-4679.1065 22550651

[33] Choi JK, Yu U, Kim S, Yoo OJ (2003). Combining multiple microarray studies and modeling interstudy variation. Bioinformatics.

[34] Tseng GC, Ghosh D, Feingold E (2012). Comprehensive literature review and statistical considerations for microarray meta-analysis. Nucleic acids research.

[35] Wang X (2012). An R package suite for microarray meta-analysis in quality control, differentially expressed gene analysis and pathway enrichment detection. Bioinformatics.

[36] Sacks FM (2009). Comparison of weight-loss diets with different compositions of fat, protein, and carbohydrates. The New England journal of medicine.

[37] Johansson LE (2012). Differential gene expression in adipose tissue from obese human subjects during weight loss and weight maintenance. Am J Clin Nutr.

[38] Larsen TM (2010). The Diet, Obesity and Genes (Diogenes) Dietary Study in eight European countries–a comprehensive design for long-term intervention. Obesity reviews: an official journal of the International Association for the Study of Obesity.

[39] Montastier E (2015). System model network for adipose tissue signatures related to weight changes in response to calorie restriction and subsequent weight maintenance. PLoS computational biology.

[40] Alligier M (2012). Subcutaneous adipose tissue remodeling during the initial phase of weight gain induced by overfeeding in humans. The Journal of clinical endocrinology and metabolism.

[41] Arner E (2012). Adipose tissue microRNAs as regulators of CCL2 production in human obesity. Diabetes.

[42] Irizarry RA (2003). Exploration, normalization, and summaries of high density oligonucleotide array probe level data. Biostatistics.

[43] Gautier L, Cope L, Bolstad BM, Irizarry R (2004). A. affy–analysis of Affymetrix GeneChip data at the probe level. Bioinformatics.

[44] Van Norstrand DW (2007). Molecular and functional characterization of novel glycerol-3-phosphate dehydrogenase 1 like gene (GPD1-L) mutations in sudden infant death syndrome. Circulation.

[45] Birley AJ (2009). ADH single nucleotide polymorphism associations with alcohol metabolism *in vivo*. Human molecular genetics.

[46] Kelly TJ, Souza AL, Clish CB, Puigserver P (2011). A hypoxia-induced positive feedback loop promotes hypoxia-inducible factor 1alpha stability through miR-210 suppression of glycerol-3-phosphate dehydrogenase 1-like. Molecular and cellular biology.

[47] Valdivia CR, Ueda K, Ackerman MJ, Makielski JC (2009). GPD1L links redox state to cardiac excitability by PKC-dependent phosphorylation of the sodium channel SCN5A. American journal of physiology. Heart and circulatory physiology.

[48] Wood IS, de Heredia FP, Wang B, Trayhurn P (2009). Cellular hypoxia and adipose tissue dysfunction in obesity. The Proceedings of the Nutrition Society.

[49] Lee YS (2014). Increased adipocyte O2 consumption triggers HIF-1alpha, causing inflammation and insulin resistance in obesity. Cell.

[50] Zhang X (2010). Adipose tissue-specific inhibition of hypoxia-inducible factor 1{alpha} induces obesity and glucose intolerance by impeding energy expenditure in mice. The Journal of biological chemistry.

[51] Halberg N (2009). Hypoxia-inducible factor 1alpha induces fibrosis and insulin resistance in white adipose tissue. Molecular and cellular biology.

[52] Krishnan J (2012). Dietary obesity-associated Hif1alpha activation in adipocytes restricts fatty acid oxidation and energy expenditure via suppression of the Sirt2-NAD+ system. Genes & development.

[53] Jiang C (2011). Disruption of hypoxia-inducible factor 1 in adipocytes improves insulin sensitivity and decreases adiposity in high-fat diet-fed mice. Diabetes.

[54] Magenta A, Greco S, Gaetano C, Martelli F (2013). Oxidative stress and microRNAs in vascular diseases. International journal of molecular sciences.

[55] Liu SC (2014). CTGF increases vascular endothelial growth factor-dependent angiogenesis in human synovial fibroblasts by increasing miR-210 expression. Cell death & disease.

[56] Winnier DA (2015). Transcriptomic identification of ADH1B as a novel candidate gene for obesity and insulin resistance in human adipose tissue in Mexican Americans from the Veterans Administration Genetic Epidemiology Study (VAGES). PloS one.

[57] Levey DJ (2004). The evolutionary ecology of ethanol production and alcoholism. Integrative and comparative biology.

[58] Heinonen S (2015). Impaired Mitochondrial Biogenesis in Adipose Tissue in Acquired Obesity. Diabetes.

[59] Hwang PH, Lian L, Zavras AI (2012). Alcohol intake and folate antagonism via CYP2E1 and ALDH1: effects on oral carcinogenesis. Medical hypotheses.

[60] Manolio TA (2009). Finding the missing heritability of complex diseases. Nature.

[61] Lee SH, Wray NR, Goddard ME, Visscher PM (2011). Estimating missing heritability for disease from genome-wide association studies. American journal of human genetics.

[62] Despres JP, Lemieux I (2006). Abdominal obesity and metabolic syndrome. Nature.

[63] Guilherme A, Virbasius JV, Puri V, Czech MP (2008). Adipocyte dysfunctions linking obesity to insulin resistance and type 2 diabetes. Nature reviews. Molecular cell biology.

[64] Greenberg AS, Obin MS (2006). Obesity and the role of adipose tissue in inflammation and metabolism. The American journal of clinical nutrition.

[65] Wellen KE, Hotamisligil GS (2003). Obesity-induced inflammatory changes in adipose tissue. The Journal of clinical investigation.

