# Supplementary material for: A Systems Genetics Approach Identified GPD1L and its Molecular Mechanism for Obesity in Human Adipose Tissue
Source: Sci Rep. 2017 May 11;7:1799. doi: 10.1038/s41598-017-01517-6 (PMC5431993; doi:10.1038/s41598-017-01517-6)
Supplement: Supplementary file 2 — Supplementary info [file 41598_2017_1517_MOESM2_ESM.doc]

**SUPPLEMENTARY DATA**

**A Systems Genetics Approach Identified *GPD1L* and its Molecular Mechanism for Obesity in Human Adipose Tissue**

Hao He^1^, Dianjianyi Sun^2^, Yong Zeng^1^, Ruifeng Wang^1^, Wei Zhu^1^, Shaolong Cao^3^, George A. Bray^4^,Wei Chen^5^, Hui Shen^1^, Frank M. Sacks^6^, Lu Qi^2,4,7^, Hong-wen Deng^1*^

**Supporting Document/Data**

Supplementary Figure S1. The topological overlap matrix (TOM) plot of coexpression network identified from gene expression profiling in GSE32512.

Supplementary Figure S2. Scatter plot of Gene Significance (GS) for obesity vs. Module Membership (MM) in the network Yellow module. There was a highly significant correlation between GS and MM in this module. Gene *GPD1L* had the largest connectives in the Yellow module.

Supplementary Figure S3. The network of miRNA-mRNA interations with FDR < 0.05.

Supplementary Figure S4. Forest plot of *GPD1L* expression across four datasets. The x-axis was the standardized mean difference between obese and non-obese subjects. Note that *GPD1L* was not measured in the GSE25401.

Supplementary Figure S5. The relationship of *GPD1L* expression with HOMA-IR. HOMA-IR values were log transformed to minimize the issue of non-normality.

Supplementary Table S1. Characteristics of all the gene expression profiles in present study

Supplementary Table S2. Association of the Yellow module genes with nine metabolic traits

Supplementary Table S3. Gene ontology enrichment analysis of the Yellow module

Supplementary Table S4. Hub genes assessed by Mouse Genome Informatics (MGI)

Supplementary Table S5. Results of meta-analysis of the Yellow module genes using Fisher's methods

Supplementary Table S6. Results of meta-analysis of the Yellow module genes using effect size combined methods. Note that 66 overlapped genes with significant results (FDR < 0.05) in both meta-analysis methods were highlighted.


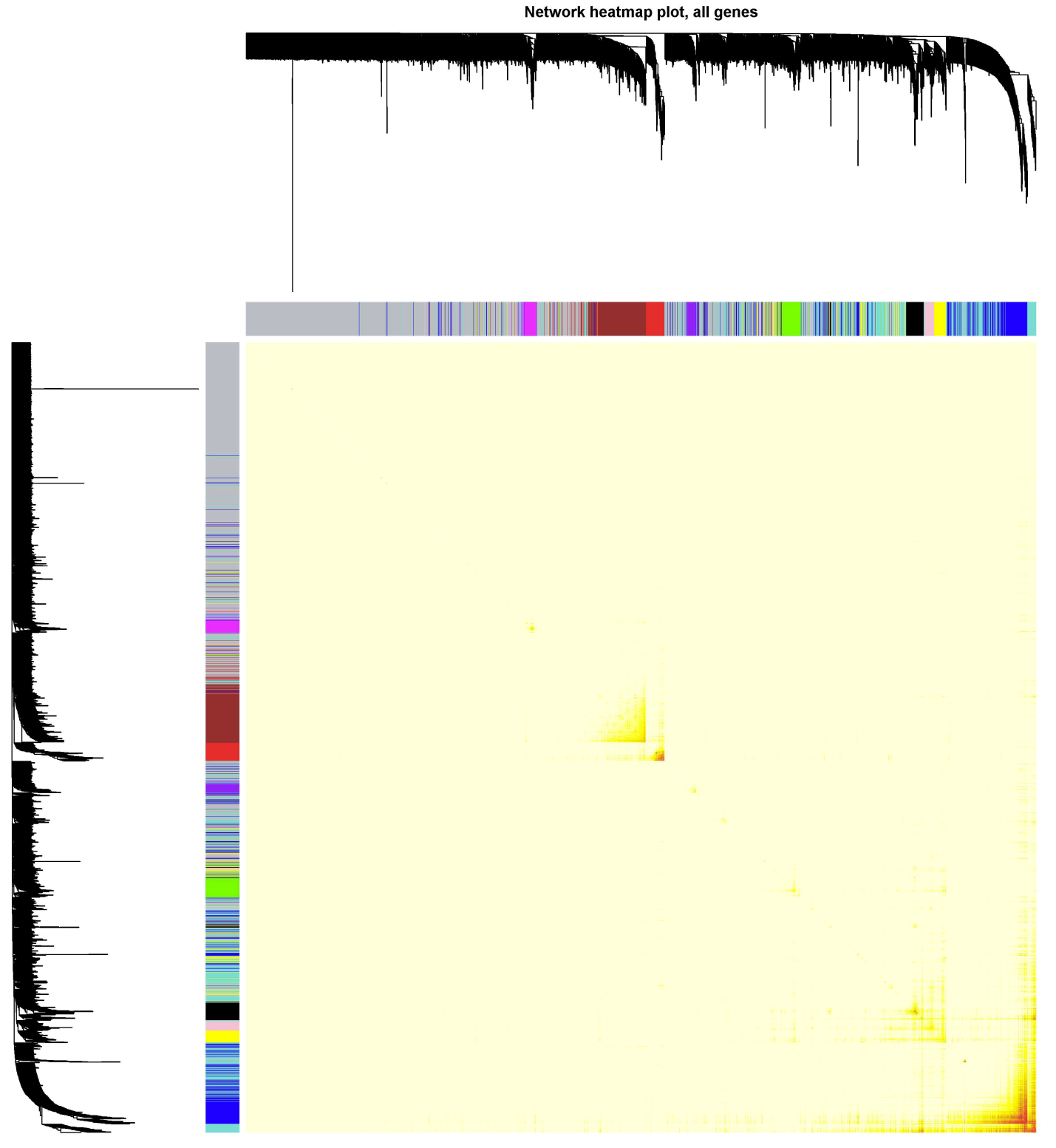


Supplementary Figure S1.


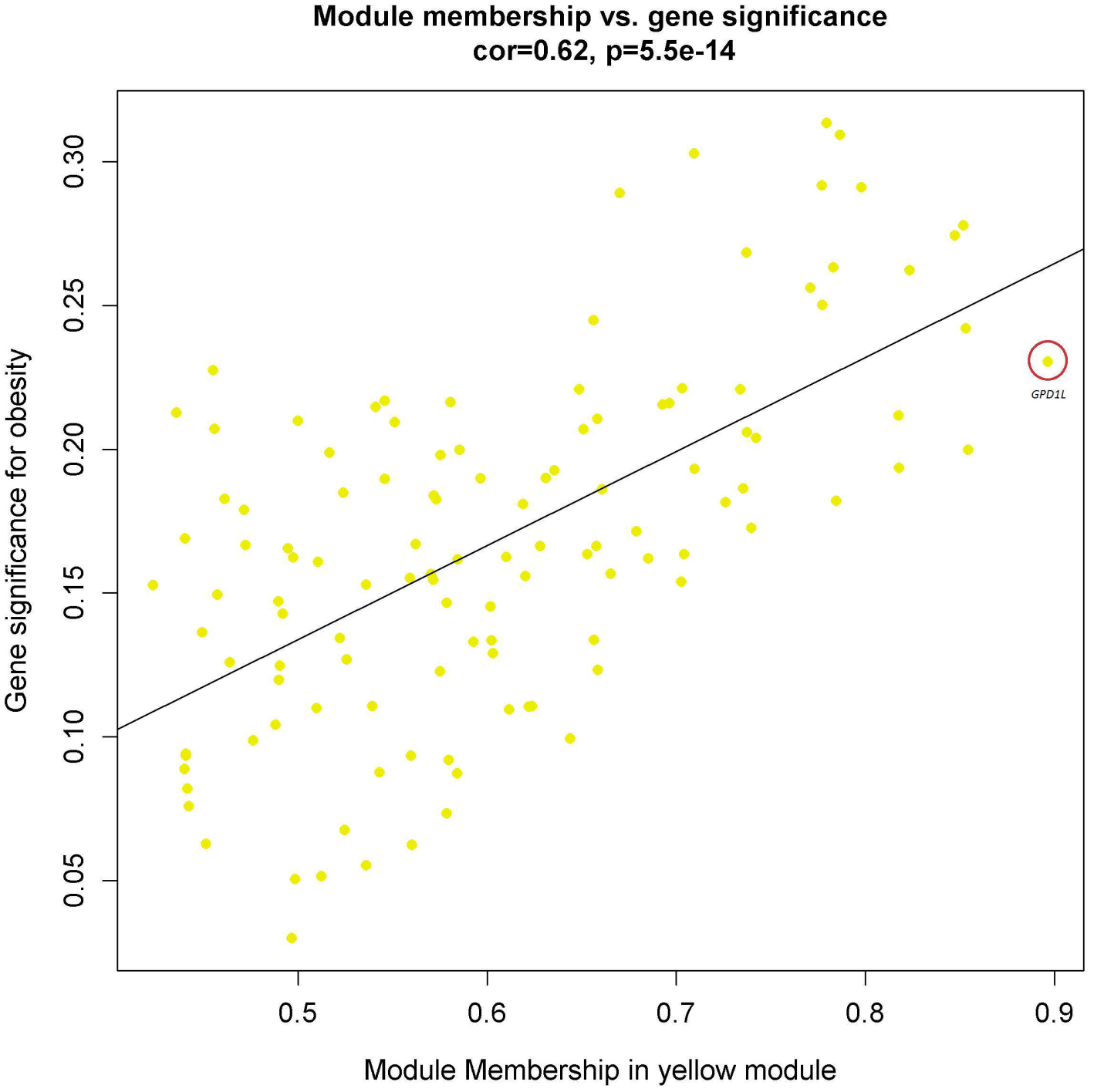


Supplementary Figure S2.


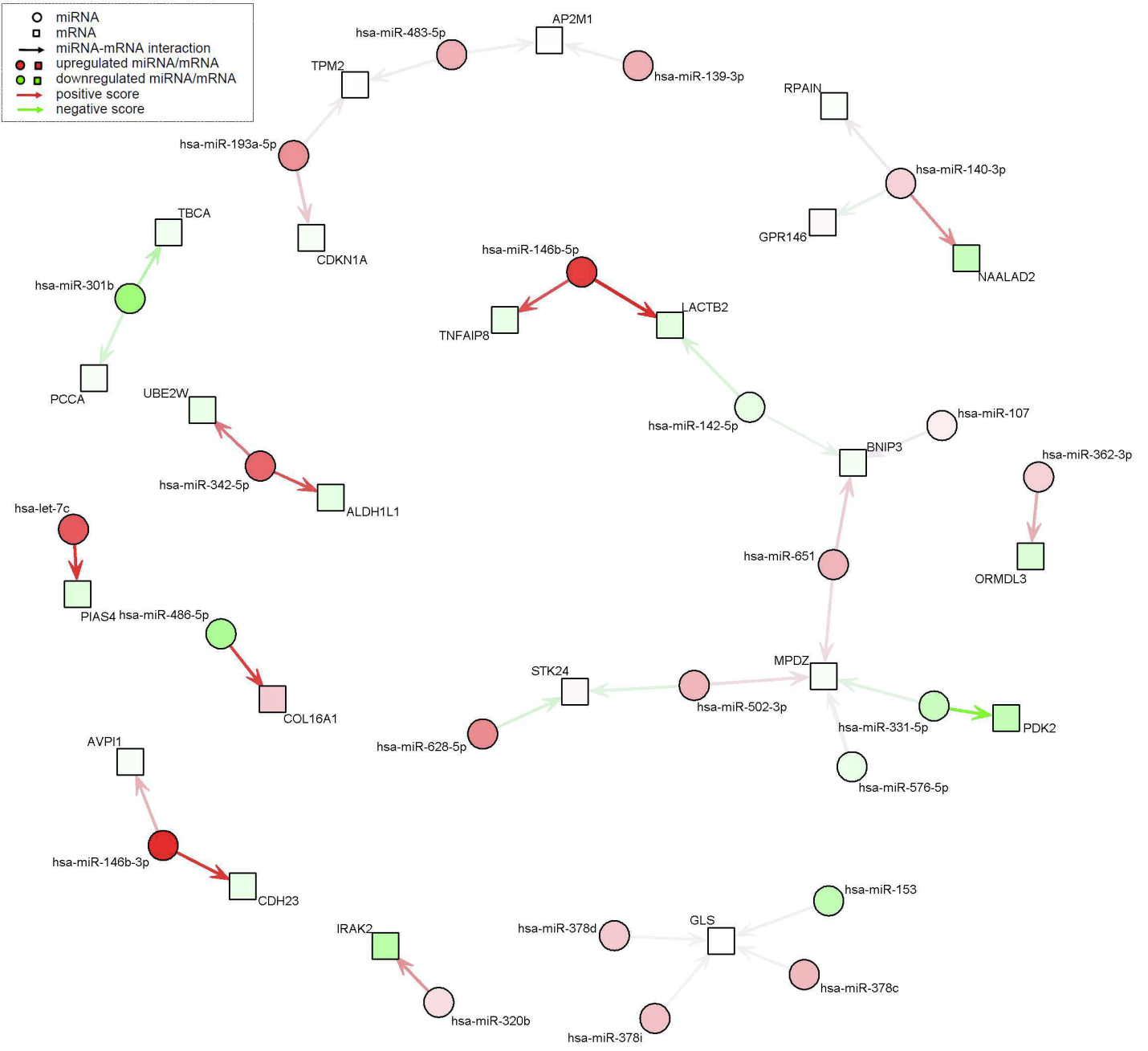


Supplementary Figure S3.


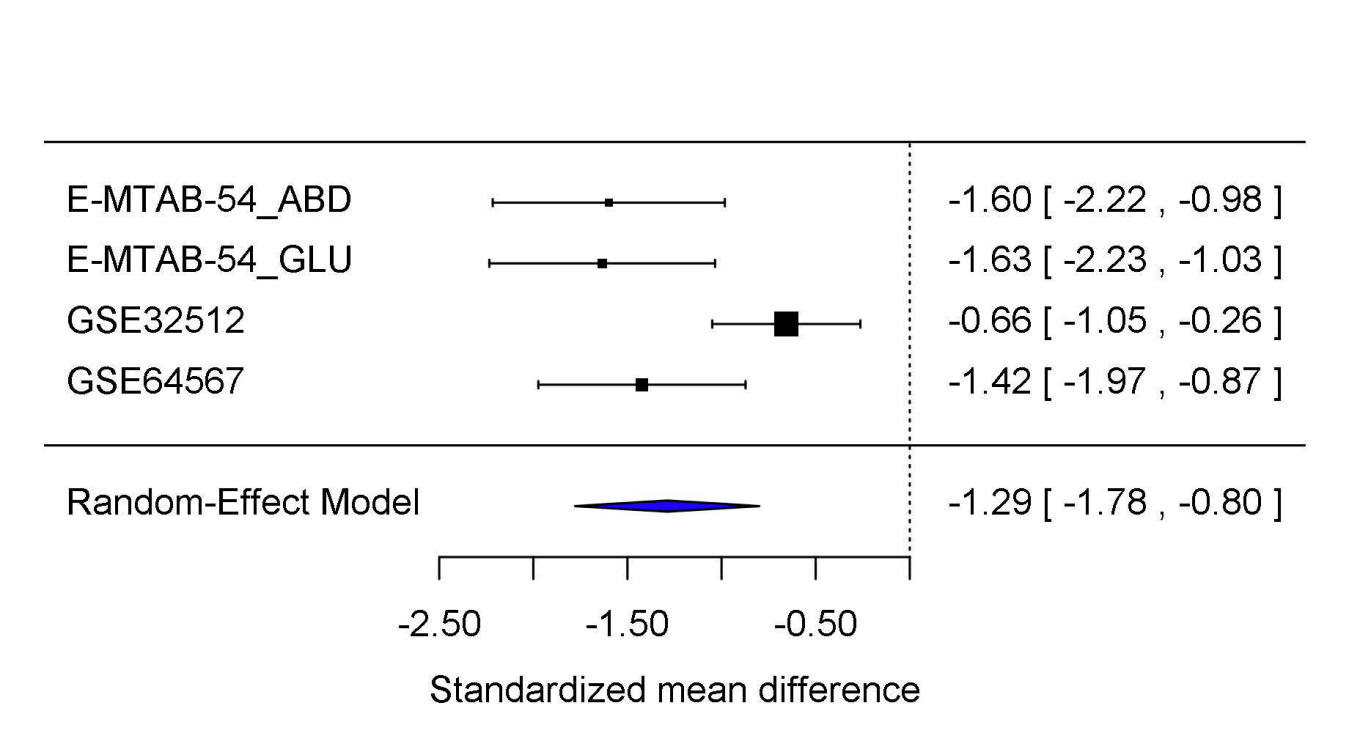


Supplementary Figure S4.


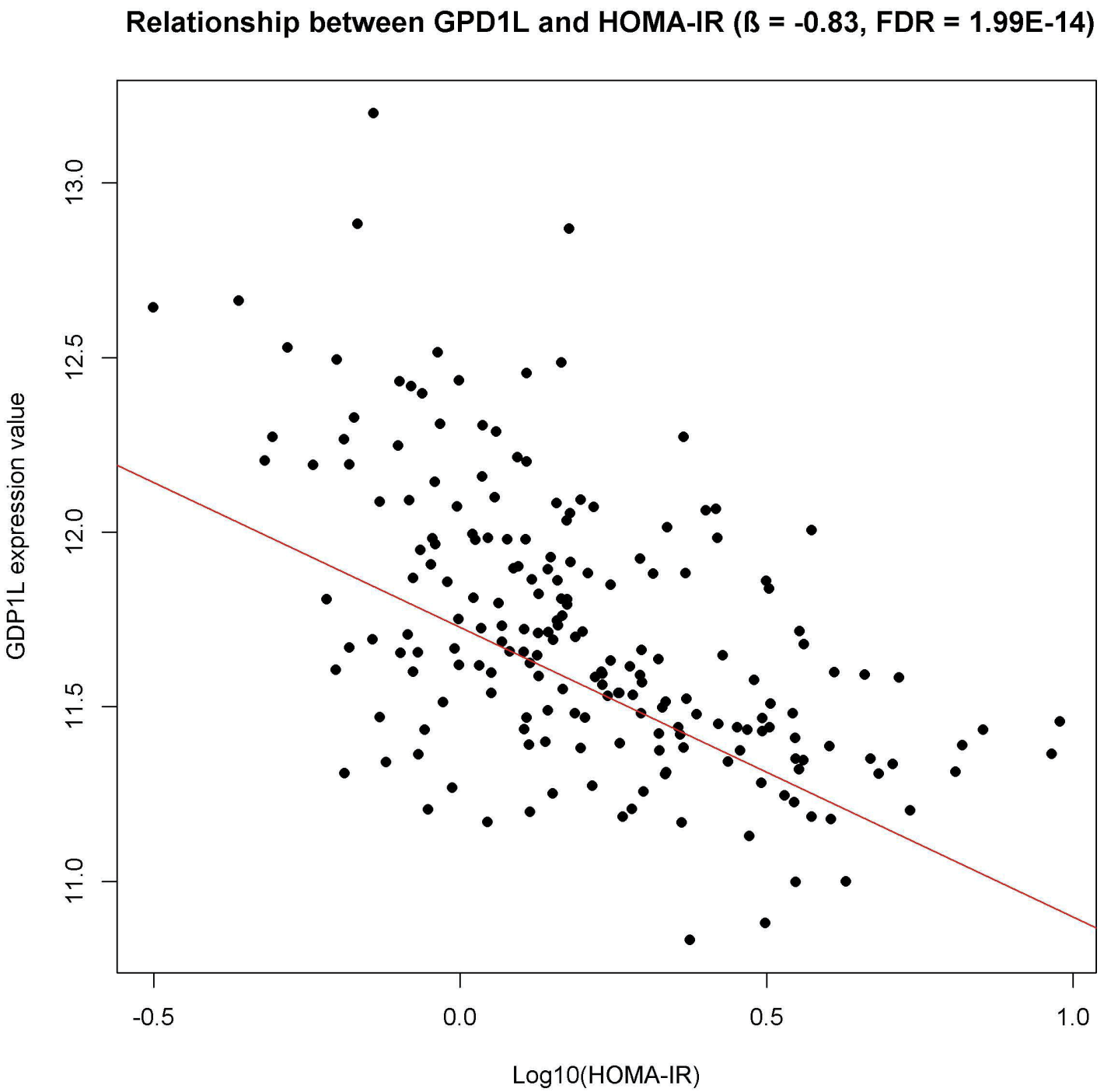


Supplementary Figure S5.
